# P068 Beyond thrombosis: acute ischaemic stroke unmasking large vessel vasculitis/giant cell arteritis

**DOI:** 10.1093/rap/rkag113.098

**Published:** 2026-11-03

**Authors:** Calvin Han Jun Tan, Sarah Loughrey

**Affiliations:** Department of Rheumatology, Craigavon Area Hospital, Southern Health and Social Care Trust, Craigavon, United Kingdom; Department of Rheumatology, Craigavon Area Hospital, Southern Health and Social Care Trust, Craigavon, United Kingdom

## Abstract

**Introduction:**

Giant cell arteritis (GCA) is the commonest large vessel vasculitis (LVV) in adults aged over 50 years and constitutes a rheumatological emergency due to the risk of irreversible ischaemic complications. Although visual loss is the classical presentation, cerebrovascular events are rare but potentially devastating, with posterior circulation strokes occurring disproportionately in GCA. Delayed recognition may result in significant morbidity despite timely glucocorticoid therapy. This case highlights a case of GCA complicated by acute ischaemic stroke, illustrating the diagnostic importance, importance of early multidisciplinary assessment, and evolving management strategies to optimise neurological and vascular outcomes in this high-risk patient group.

**Case description:**

An 80-year-old woman presented via an emergency thrombolysis pathway with acute limb weakness, gait disturbance, and dysphasia. Her past medical history included anaemia, uterovaginal prolapse, and essential thrombocytosis. Thrombolysis was not administered as she presented outside the treatment window. Initial CT angiography demonstrated a left vertebral artery dissection, and she was admitted under the stroke team with a presumed posterior circulation stroke.

During admission, it was noted that she had persistently elevated inflammatory markers (ESR at 100s mm/h and CRP at 100s mg/L) of unknown aetiology for the preceding three months. Despite this, she reported no cranial symptoms suggestive of giant cell arteritis (GCA) and no features of polymyalgia rheumatica (PMR). Given the unexplained inflammatory response, urgent temporal artery ultrasound and FDG PET-CT were requested. Temporal artery ultrasound demonstrated mild bilateral halo signs involving the frontal branches, prompting initiation of prednisolone 40 mg daily. FDG PET-CT, performed two days after commencing glucocorticoid therapy, demonstrated extensive large-vessel vasculitis involving the entire aorta, the major branches of the aortic arch, including both vertebral arteries, confirming the diagnosis of large-vessel GCA.

Following discharge, she remained clinically well initially under rheumatology follow-up. However, two weeks later she re-presented with acute speech disturbance. Repeat CT angiography demonstrated complete stenosis of the left vertebral artery with proximal V4 segment occlusion, together with progressive inflammatory wall thickening involving the brachiocephalic and subclavian arteries, in keeping with active vasculitis. She subsequently disclosed that she had discontinued prednisolone after one week because of vomiting.

She was treated with three days of intravenous methylprednisolone followed by prednisolone 60 mg daily. Subcutaneous tocilizumab was planned as steroid-sparing therapy, but its initiation was delayed because of gastroenteritis and a concurrent norovirus outbreak on the ward. She subsequently made a good neurological and clinical recovery with no further vascular events.

**Discussion:**

This case highlights an atypical presentation of GCA with extensive large-vessel involvement manifesting as firstly vertebral dissection, then ischaemic stroke. GCA is the most common systemic vasculitis in older adults and classically presents with cranial symptoms such as headache, scalp tenderness, jaw claudication and visual disturbance. However, up to one-third of patients demonstrate predominantly extracranial large-vessel disease, which may occur in the absence of typical cranial or polymyalgia rheumatica symptoms. In this case, the persistent unexplained elevation of inflammatory markers for three months preceding presentation provided a clue to an underlying inflammatory process, but not specific.

The initial presentation with dysphasia, gait disturbance and limb weakness prompted acute stroke assessment, with CT angiography demonstrating left vertebral artery dissection. Although vertebral artery involvement can be related to possible GCA, it remains an uncommon manifestation and can result in posterior circulation ischaemia. In addition, she has lack of common GCA symptoms. The coexistence of markedly elevated inflammatory markers and vascular abnormalities raised suspicion for possible vasculitis. Temporal artery ultrasound demonstrated bilateral halo signs, supporting the diagnosis, while FDG PET-CT subsequently confirmed extensive large-vessel vasculitis involving the aorta and major branches, including the vertebral arteries.

The subsequent cerebrovascular event occurred shortly after discontinuation of prednisolone, highlighting the aggressive nature of large-vessel GCA and the importance of sustained immunosuppression. Repeat vascular imaging demonstrated progression from vertebral artery dissection to complete stenosis and occlusion, consistent with ongoing active vascular inflammation. This significant event strongly suggests disease relapse due to interruption of treatment rather than treatment failure.

Management required escalation to high dose glucocorticoids therapy, with plans for tocilizumab as a steroid-sparing and disease-modifying agent. Fortunately, the patient achieved clinical recovery, successful rehabilitation and biochemical remission. This case emphasises the need for heightened awareness of possible link between stroke and unexplained systemic inflammation with GCA.

**Key learning points:**

• This case provides several important learning points.

• Stroke is a rare complication from GCA. However, GCA should be considered in older patients presenting with stroke, particularly posterior circulation stroke, when inflammatory markers are disproportionately elevated without an alternative explanation. The absence of classical GCA symptoms does not exclude the diagnosis, as we know large vessel vasculitis can present heterogeneously. It may present solely with constitutional features, persistently raised inflammatory markers or vascular complications.

• Secondly, vertebral artery involvement is an important but under-recognised manifestation of GCA. Inflammation of the vertebral arteries can result in stenosis, occlusion, dissection and recurrent posterior circulation ischaemic events. Recognition of this association is crucial because standard stroke pathways may not identify the underlying inflammatory cause. Careful review of vascular imaging and inflammatory markers can provide important diagnostic clues.

• Thirdly, multimodal imaging plays a key role in the diagnosis of large-vessel vasculitis. Temporal artery ultrasound remains a valuable first-line investigation and, in this case, demonstrated bilateral halo signs despite the absence of typical cranial symptoms. FDG-PET CT further confirmed widespread aortic and branch vessel involvement. The complementary use of vascular ultrasound, CT angiography and PET imaging facilitated accurate diagnosis and risk stratification.

• Fourthly, treatment adherence is critical in GCA. This case demonstrates how interruption of glucocorticoid therapy for only one week was associated with disease progression and further cerebrovascular ischaemia. Patients should receive counselling regarding the importance of continuing treatment and seek medical advice promptly if adverse effects such as vomiting impair medication administration.

• Finally, patients with GCA/LVV represent a high-risk subgroup who often require early consideration of steroid-sparing therapy. Tocilizumab should be considered early as it has been shown to reduce relapse rates and cumulative glucocorticoid. Close collaboration between rheumatology, stroke medicine, and radiology is essential to optimise outcomes in these complex cases.

